# Herring gulls respond to the acoustic properties of men’s voices

**DOI:** 10.1098/rsbl.2025.0394

**Published:** 2025-11-12

**Authors:** Céline M. I. Rémy, Christophoros Zikos, Laura Ann Kelley, Neeltje Janna Boogert

**Affiliations:** ^1^ AgroParisTech Campus Agro Paris-Saclay, Palaiseau, Île-de-France, France; ^2^ Université Paris-Saclay Faculté des Sciences d'Orsay, Orsay, Île-de-France, France; ^3^ Centre for Ecology and Conservation, University of Exeter, Penryn, Cornwall, UK

**Keywords:** foraging, human–wildlife conflict, playback, communication, deterrent, urban ecology

## Abstract

Due to ongoing global urbanization, some animals have settled in urban environments and rely increasingly on anthropogenic resources. One such urban adapter is the European herring gull, *Larus argentatus*, whose presence in towns has led to conflict with humans. Previous research has found gulls perceive men's shouting as a threat. We conducted a playback experiment on wild urban herring gulls in a foraging context to determine whether gulls perceive the difference between men shouting versus speaking the same words at the same volume, and whether those stimuli represented the same level of threat. Gulls reacted similarly to men shouting and speaking, as they flinched at the playback, exhibited vigilance, pecked less at the human food source and left the apparatus sooner than when exposed to robin song. However, gulls differentiated between the acoustic properties of men’s vocalizations, as they flew away from men shouting but walked away from men speaking. When attempting to deter gulls from exploiting anthropogenic resources, talking might stop them from foraging, but shouting is more effective at making them flee.

## 1. Introduction

Urban populations of animals are more tolerant of humans than their rural counterparts, demonstrated by their reduced escape behaviour [1,2], boldness [3] and lower physiological stress response to humans [4]. Human food provisioning may facilitate adaptation to urban environments [5]. However, anthropogenic pressures such as habitat fragmentation and pollution through water, air, soil, light or noise [2,6] can cause rapid population-level modifications in behaviour [7]; for example, bobcats (*Lynx rufus*) and coyotes (*Canis latrans*) have larger home ranges and higher night-time activity in urban habitats [8].

With increased proximity to urban areas, comes more frequent interactions with humans, but humans vary in their attitude towards wildlife in cities, and urban wildlife can respond accordingly [9]. For example, African elephants (*Loxodonta africana*) and Poeppig’s woolly monkeys (*Lagothrix poeppigii*) that live near human settlements differentiate between dangerous, neutral and friendly humans by adapting their behaviour to flee from or seek out the respective groups [10,11]. Some corvid species (Corvidae) and pigeons (*Columba livia*) are able to recognize individual humans or humans with similar characteristics (e.g. wearing particular clothing) [9]. By relying on human cues, urban animals can adapt their behaviour to the nature of different human interactions. It is possible, however, that urban animals may only be coping rather than thriving in urban environments [12,13], which may translate into reduced reproduction, premature ageing and/or increased mortality [14–16].

One species of bird that has rapidly spread in urban habitats is the European herring gull (*Larus argentatus*). Herring gulls have colonized most of the British Isles in the past century, and now the majority of the UK’s herring gull population nests in urban areas [17]. Gulls’ presence in urban spaces has led to conflict with humans due to their noisiness, messiness and foraging [18]. Many urban gulls forage at landfill sites [19], but others forage within towns and cities, thus interacting with a larger human population [20]. These gulls are attracted to anthropogenic objects that may contain food and/or have been handled by humans [21]. Gulls also use human behavioural cues to decide when to steal food [21,22]. Gulls’ food stealing is exacerbated by people feeding them, accidentally or not, despite city councils running awareness campaigns requesting people to not feed the gulls [23].

Certain human behaviours can dissuade gulls from stealing food. Simply approaching a gull induces a strong flight response [24]. Gulls, like many other species, are aversive to human gaze [25], and therefore less likely to approach a person with food if they keep eye contact with the gull. Gulls are also sensitive to auditory cues and respond with similar urgency to conspecific alarm calls and men shouting [26]. Furthermore, the louder the stimulus, the more urgently gulls flee [27]. However, it is not known whether gulls are averse to human vocalizations in general, or whether they respond to the acoustic properties of human vocalizations regardless of volume; i.e. are gulls more wary of a human shouting than of a human speaking?

This study quantified the behavioural responses of wild urban herring gulls, foraging in coastal towns, to the playbacks of three auditory stimuli at the same volume: a man shouting, a man speaking and robin song. Robin song, a familiar and non-threatening sound to the gulls, served as a negative control and we used men’s voices as in [26]. We predicted that gulls would perceive a man shouting and speaking both as threats, but that a man speaking would represent a lower threat level than a man shouting at the same playback volume.

## 2. Methods

### (a) Test subjects

We tested 61 wild herring gulls across nine towns in Cornwall, Southwestern England, between 24 February and 14 March 2025. The trials were conducted from 08.30 to 16.30, during the non-breeding season, when the weather was dry and not windy.

The targeted individuals were in stationary positions either on objects up to 2 m above ground level or on utility poles or lamp posts. The gulls were tested in 52 different locations separated by at least 50 m (map in electronic supplementary material, figure S1). When conducting trials at the same or nearby locations, we visually tracked previously tested gulls to limit the risk of pseudo-replication within that group of trials. To further limit that risk, we did not return to the same neighbourhoods after having conducted trials there.

### (b) Recordings

We used three different treatments in our playback experiment: man shouting, man speaking and European robin (*Erithacus rubecula*) song, referred to as ‘Shouting’, ‘Speaking’ and ‘Robin,’ respectively. We obtained five recordings per treatment, with each recording looped to generate a 30 s track.

For the Shouting and Speaking treatments, we recorded five British male volunteers who were not associated with the project. They recorded themselves saying ‘No! Stay away! That’s my food, that’s my pasty!’ first in a ‘shouting’ voice, and then a second time in a neutral ‘speaking’ voice. The recordings were made on mobile phones and sent as opus files, which we converted to mp3. Five 10-s-long robin song recordings were sourced from the online Xeno-Canto library [28] (mp3 files XC403639, XC631602, XC698392, XC945925 and XC965086).

Recordings were edited in Audacity® version 3.7.2.0 to normalize each track to 60 dB peak amplitude (dB A weighting; 20 μPA reference value) and remove background noise. These values reflect what gulls would experience under natural conditions [29] and allowed us to compare results with [26]. Shouting and speaking recordings were looped five times with several seconds of silence between each repetition to obtain a 30 s track. Robin song recordings were looped three times to obtain a track of the same duration.

The fundamental frequency of each recording was obtained using the Spectrum plot in Audacity (electronic supplementary material, table S1). A Kruskal–Wallis test confirmed all three treatments were significantly different from each other in terms of fundamental frequencies (*χ*^2^ = 11.60, d.f. = 2, *p* = 0.003).

### (c) Experimental protocol

We placed the speaker (FOXPRO^®^ Fury GX7 Digital Game Caller) in a plastic shopping bag (450 × 420 mm) to avoid gull wariness. Before testing, we used a sound meter app (rootApps, version 3.1.6) to adjust the speaker volume so that the recording could be heard at 60 dB from 150 cm away while the speaker was in the bag. We filled a clear plastic box (155 × 90 × 70 mm) a third of the way with chips (*ca* 250 g) and closed it to prevent the gulls from accessing the chips.

**Figure 1.**
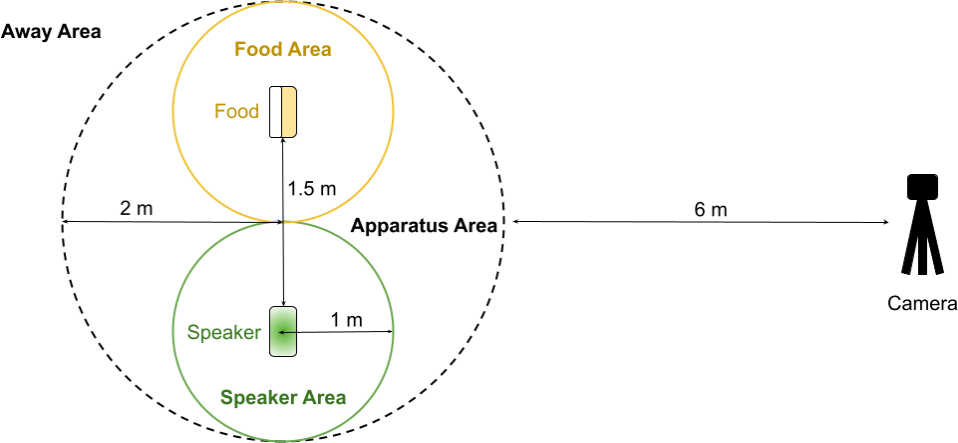
Diagram of the apparatus.

Upon finding a gull to test, one experimenter walked a few steps towards the gull and placed the bag with the speaker at their feet, while the observer retreated 8 m and placed a GoPro (GoPro Hero 3+) on a tripod 15 cm above the ground. The experimenter shook the box with chips to get the gull’s attention before placing the box upside down 150 cm away from the speaker (figure 1). The experimenter then joined the observer behind the camera. Once the chips were placed on the ground, the observer started recording and monitored the gull’s actions via live feed on their phone (GoPro Quik: Video Editor™, version 13.13.1). This allowed the experimenters to avoid looking directly at the gull [25].

Once the gull was within the apparatus area (figure 1), the experimenter started the randomly chosen 30 s playback with a remote controller, marking the beginning of the trial. The observer then waited 60 s before ending the video recording. The chips and bag were then collected and the trial location recorded on Google Maps™ before moving to a different location, or a few metres away if the tested gull had left and another untested gull was nearby.

### (d) Behavioural analysis

We imported the videos into BORIS version 8.25 [30] and for each focal gull recorded flinching (a sudden movement backwards), duration of vigilance (scanning for danger with the head level and neck extended), pecking at the food or the bag with the speaker, duration of stay near the apparatus and how/if the gull left at the end of the trial (electronic supplementary material, table S2). Electronic supplementary material, video S1 provides an example trial for each treatment.

We recorded whether the gull was alone or accompanied (‘group’). In instances where multiple gulls were present, we focused on the gull that was first to engage with the apparatus and not chased away by another gull within the first 10 s of the trial. For each trial, we recorded the focal gull’s latency to approach the apparatus, i.e. the duration between the gull landing on the ground (or taking a step towards the apparatus if it was already on the ground) and entering the apparatus area (figure 1). If the gull landed within the apparatus area, latency to approach was set to 1 s. We then exported the duration (if applicable) and frequency of each behaviour to our datasheet.

All video annotations were performed by the same person. To check for observer bias, the experimenter and the observer both annotated six trials (10% of the trials). The intraclass correlation coefficient (ICC) between the two observers for the five behaviours of interest was computed using the icc function of the irr package [31], using a two-way random-effects model, single rating and absolute agreement. We found an ICC of 0.97 with *p* < 0.001, indicating a high level of agreement between observers.

### (e) Statistical analysis

All analyses were conducted using R statistical software version 4.4.0 [32]. Two outliers were identified using Grubbs' test from the outliers package [33] and removed as they were caused by errors in coordination between experimenters. Zero-inflated gamma and ordered beta generalized linear mixed models (GLMM) were fitted with the glmmTMB package [34]. Poisson generalized linear models (GLM) were fitted using the glm function in lme4 [35] and multinomial mixed models (MMM) were fitted using the mclogit package [36]. Diagnostic plots were generated using the DHARMa package [37]. Graphs were made in ggplot2 [38].

To study ‘flinching’, we used a Poisson GLM with ‘treatment’ (Shouting, Speaking, Robin), ‘latency to approach’ and ‘group’ (gull alone or accompanied) as the predictors. We used the contrasts between treatments to determine their significance and adjusted *p*-values using the Tukey method. We used zero-inflated gamma GLMMs to study ‘duration of vigilance’ and ‘pecking,’ respectively, with fixed effects ‘treatment’, ‘latency to approach’, their interaction and ‘group’. We included ‘town’ and ‘playback frequency’ as random effects to control for pseudo-replication.

To test whether the playback treatment affected gulls’ duration of stay, we calculated the proportion of time the gull stayed within the apparatus area. As some gulls stayed for the entire duration of the trial, an ordered beta distribution defined for the closed [0,1] interval was required to analyse these data (‘duration of stay’). We used an ordered beta GLMM and an MMM to study ‘duration of stay’ and ‘end of trial’, respectively, with ‘treatment’, ‘latency to approach’, their interaction (only for duration of stay, as the model for ‘end of trial’, which included the interaction, failed to converge) and ‘group’ as fixed effects and ‘town’ and ‘playback frequency’ as random effects. We used a Welch two-sample *t*‐test to determine whether responses to shouting and speaking were different for duration of stay. All data and code for statistical analyses can be found in the electronic supplementary material.

## 3. Results

None of the gulls flinched at the Robin playbacks, which was significantly less often than in response to both the Shouting (GLM contrasts for Robin—Shouting, estimate = −0.545, 95% CI = [−0.877, −0.213], *z* ratio = −3.220, *p* = 0.004) and Speaking treatments (GLM contrasts for Robin—Speaking, estimate = −0.486, 95% CI = [−0.797, −0.174], *z*-ratio = −3.058, *p* = 0.006), while there was no significant difference in whether gulls flinched between the Shouting and Speaking treatments (GLM contrasts for Shouting—Speaking, estimate = 0.060, 95% CI = [−0.365, 0.484], *z* ratio = 0.275, *p* = 0.960). Additionally, gulls exposed to the Shouting and Speaking playbacks were significantly more vigilant (GLMM conditional model for Shouting, estimate = 0.768, 95% CI = [0.029, 1.507], *z* = 2.037, *p* = 0.042; Speaking, estimate = 0.744, 95% CI = [−0.002, 1.491], *z* = 1.954, *p* = 0.051) than those exposed to the Robin playbacks. Gulls exposed to the Shouting or Speaking playbacks were also significantly less likely to continue pecking (GLMM zero-inflation model for Shouting, estimate = 3.187, 95% CI = [0.887, 5.487], *z* = 2.716, *p* = 0.006; Speaking, estimate = 3.008, 95% CI = [0.662, 5.354], *z* = 2.513, *p* = 0.012).

**Figure 2.**
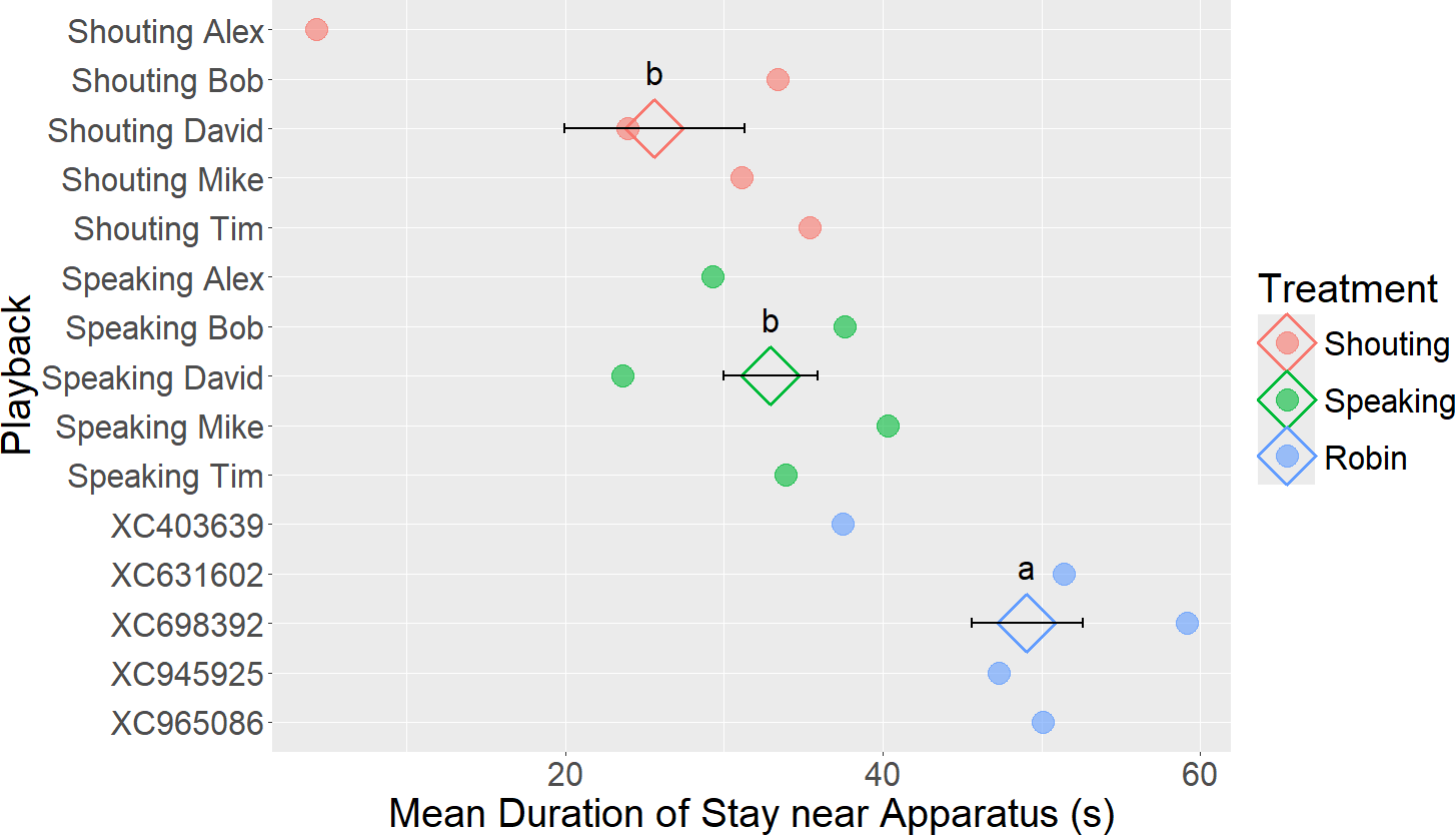
Mean duration of stay near the apparatus during the trial per playback ID and treatment (each dot represents the mean duration of stay calculated for the 3–5 individual gulls exposed to the playback stimulus ID on the *y*-axis). Diamonds indicate the centroid for each treatment as well as the standard deviation. Gulls subjected to the Shouting and Speaking treatments left sooner than those subjected to the Robin treatment. Significant differences (*p* < 0.05) between treatments are shown by different letters (a, b).

A total of 14 gulls (23% of all gulls tested) stayed within the apparatus area for the whole 1 min trial. There were two instances where a gull flew away from the apparatus but returned before the end of the trial, and multiple instances of gulls wandering in and out of the apparatus area. Gulls exposed to the Shouting and Speaking playbacks stayed near the apparatus for significantly shorter periods of time than those exposed to the Robin treatment (GLMM for Shouting, estimate = −1.700, 95% CI = [−2.647, −0.753], *z* = −3.520, *p* = 0.001; Speaking, estimate = −1.110, 95% CI = [−2.034, −0.186], *z* = −2.353, *p* < 0.001; figure 2). There was, however, no significant difference in duration of stay between the Shouting and Speaking treatments (Welch two-sample *t*‐test, *t* = −0.837, df = 38.749, *p* = 0.408). Gulls who were quick to approach the apparatus stayed significantly longer, regardless of the treatment (GLMM for latency to approach, estimate = −0.066, 95% CI = [−0.135, 0.003], *z* = −1.884, *p* = 0.019).

**Figure 3.**
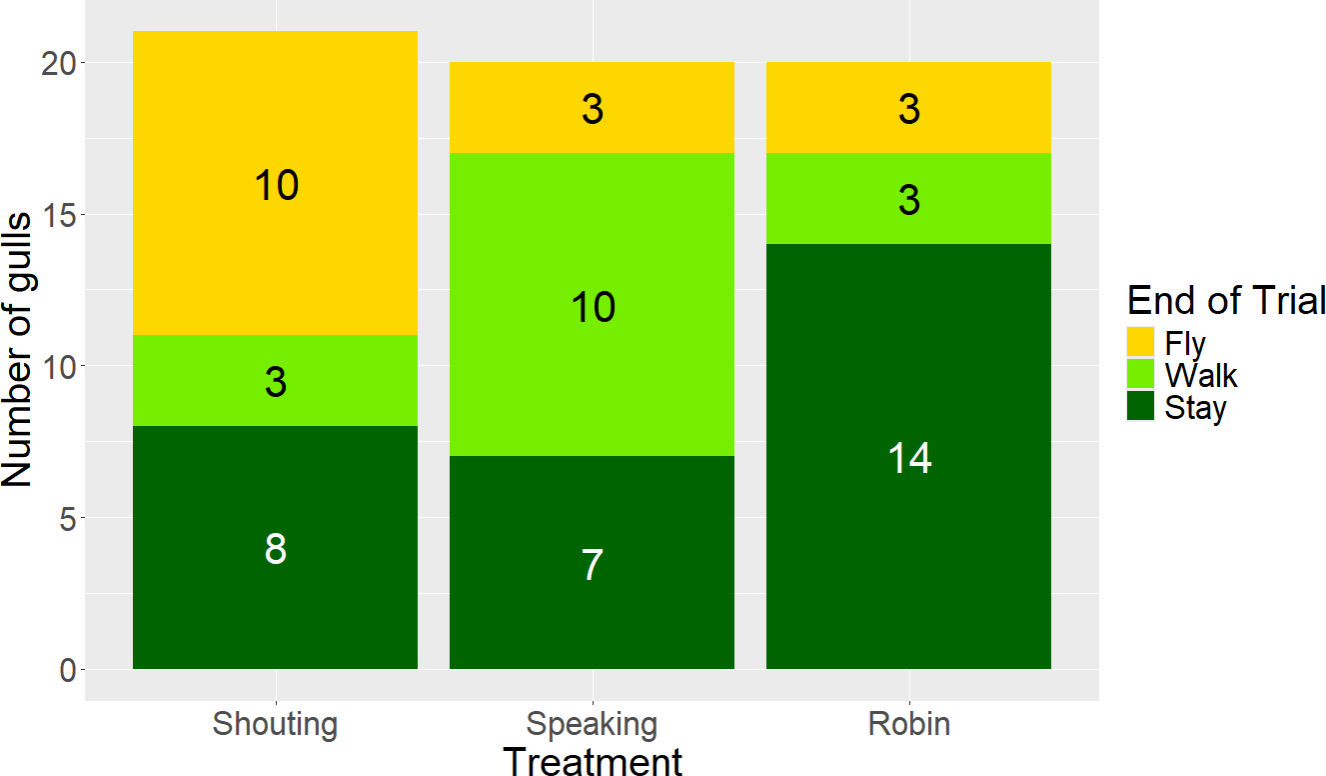
Number of gulls staying near the apparatus, flying away or walking away at the end of the trial. Of the gulls that left the apparatus before the end of the trial, gulls subjected to the Shouting treatment flew away from the apparatus, whereas those in the Speaking treatment walked away.

When gulls left before the end of the 60 s trial, those exposed to the Shouting playbacks were five times more likely to fly away, whereas gulls exposed to the Speaking playbacks were seven times more likely to walk away, as compared to those exposed to the Robin playback (MMM for fly~Shouting, odds ratio (OR) = 5.154, 95% CI = [1.063, 25.000], *z* = 2.035, *p* = 0.042; walk~Speaking, OR = 6.964, 95% CI = [1.062, 45.676], *z* = 2.022, *p* = 0.043; table 1, figure 3).

**Table 1.** Summary of the multinomial model for ‘end of trial’. Odds ratios and 95% CIs were calculated using the log-odds estimates and their standard errors. The ‘stay’ level is used as the baseline for comparing levels. Significant terms are highlighted in italics.

	odds ratio	95% CI	z	*p-*value
fly~(intercept)	0.423	[0.077, 2.322]	−0.991	0.322
*fly~treatment_shouting*	*5.154*	*[1.063, 25.000]*	*2.035*	*0.042*
fly~treatment_speaking	1.744	[0.267, 11.411]	0.581	0.561
fly~latency_approach	0.942	[0.764, 1.163]	−0.553	0.580
fly~group_yes	0.478	[0.121, 1.888]	−1.053	0.292
*walk~(intercept)*	*0.115*	*[0.014, 0.931]*	*−2.027*	*0.043*
walk~treatment_shouting	1.839	[0.207, 16.309]	0.547	0.584
*walk~treatment_speaking*	*6.964*	*[1.062, 45.676]*	*2.022*	*0.043*
*walk~latency_approach*	*1.155*	*[1.000, 1.335]*	*1.957*	*0.050*
walk~group_yes	0.606	[0.125, 2.923]	−0.624	0.532

## 4. Discussion

We found that gulls consider both a man speaking and a man shouting to be threats, as they exhibited more wariness in response to the playbacks of these stimuli than to the playbacks of robin song. Gulls subjected to the robin treatment never flinched at the playback. Gulls exposed to the shouting and speaking playbacks exhibited similar durations of vigilance, which were significantly longer than those of the gulls exposed to the robin playbacks. Individuals exposed to playbacks of male voices pecked at the speaker or the food fewer times and stayed near the apparatus for a shorter length of time than those exposed to robin song. Gulls exposed to the shouting treatment flew away, whereas the gulls exposed to the speaking treatment walked away. These findings demonstrate that gulls perceive a man shouting to be a greater threat than a man speaking. Thus, when deterring gulls from anthropogenic resources, both speaking and shouting are likely to stop a gull from foraging, but shouting is a more effective way to encourage a gull to leave an area.

Urban herring gulls are able to adapt their foraging schedule to human activity and target areas at times when human food is easily accessible, such as school playgrounds during recess [39] or town squares during lunch time [20]. This increases the number of human–gull interactions, with negative consequences for both human quality of life [18] and gull breeding success [40], such as compromised chick nutrition [41]. It is therefore important to deter gulls from approaching anthropogenic food sources, and our findings show that male human vocalizations, and shouting in particular, may help to mitigate human–gull conflict over human food.

Some studies have shown that captive wild animals such as gorillas (*Gorilla gorilla*) and grey wolves (*Canis lupus*) can discriminate between familiar and unfamiliar voices [42,43]. One study demonstrated that wild African elephants can distinguish between languages but did not test their reaction to changes in acoustic properties within a single language [44]. To our knowledge, this study is the first to test whether wild, non-captive animals perceive differences in the acoustic properties of male human voices uttering the same sentences.

Using human food to attract gulls for testing may have resulted in a bias for testing bolder, less neophobic individuals that were naturally more likely to engage with our apparatus [26]. However, one aim of this study was to investigate whether those gulls attracted to human food differentiate between shouting and speaking people. By testing the gulls that showed interest in foraging on human food in the presence of humans, we tested individuals that may be the ‘problem individuals’ willing to steal food from humans.

We expected that gulls may be more likely to engage with the apparatus in more heavily populated towns, but this was not the case, as shown by the small amount of variance explained by ‘town’ in the models. St Ives and Hayle are half as populous as Penzance and St Austell. However, a total of 14 gulls approached the apparatus in St Ives and Hayle, whereas only five did in Penzance and St Austell. The number of successful trials conducted in each town, i.e. the number of gulls willing to engage with the apparatus, could be linked to tourism. Towns with higher levels of tourism, such as St Ives and Hayle, could provide more foraging opportunities to gulls, as tourists may feed gulls and there is more food available from street vendors and people eating outdoors.

This study raises the question of whether gulls use differences in pitch or inflections of tone to discriminate between a man speaking versus shouting. Conducting a similar study incorporating female voices could give us insight into whether gulls are sensitive to pitch. It is possible that herring gulls can discriminate between genders and are more scared of one than the other, as in wild jackdaws (*Corvus monedula*) [45]. Another approach would be to compare the gulls’ reactions to human voices and artificial sounds following the same inflections and pitch as the human voices. Overall, deterring gulls from an area is likely to be more effective through shouting than speaking, although there may be subtle differences in a gull’s strength of reaction based on cues such as human vocalization pitch and intonation. Combining human behaviours known to be aversive to urban gulls, such as approaching, staring [24] and shouting (this study), is likely to provide the most effective means of deterring gulls from human food.

## Data Availability

The data and code have been provided as electronic supplementary materials. Supplementary material is available online [46].
